# Smallest known raptor tracks suggest microraptorine activity in lakeshore setting

**DOI:** 10.1038/s41598-018-35289-4

**Published:** 2018-11-15

**Authors:** Kyung Soo Kim, Jong Deock Lim, Martin G. Lockley, Lida Xing, Dong Hee Kim, Laura Piñuela, Anthony Romilio, Jae Sang Yoo, Jin Ho Kim, Jaehong Ahn

**Affiliations:** 1 Department of Science Education, Chinju National University of Education, 3 Jinnyangho-ro 369beon-gil, Jinju-si, Gyeongnam 52673 South Korea; 2 Cultural Heritage Administration, Government Complex-Daejeon, 18, Cheongsa-ro, Seo-gu, Daejon 35208 South Korea; 3 Dinosaur Trackers Research Group, University of Colorado Denver, P.O. Box 173364, Denver, CO 80217-3364 USA; 4 School of the Earth Sciences and Resources, China University of Geosciences, Beijing, 100083 China; 5 National Science Museum, 481 Daedeok-daero, Yuseong-gu, Daejeon 34143 South Korea; 6 Museo del Jurásico de Asturias MUJA (Jurassic Museum of Asturias), Colunga, E-33328 Spain; 7 School of Biological Sciences, the University of Queensland, Brisbane, Qld 4072 Australia; 8 Graduate School of Culture Technology, Korea Advanced Institute of Science and Technology, 291, Daehak-ro, Yuseong-gu, Daejeon 34141 South Korea

## Abstract

Ongoing studies of a multiple track-bearing horizons from massive excavations in the Jinju Formation (Lower Cretaceous) of South Korea have yielded a remarkable diversity of avian, non-avian dinosaur, pterosaur, crocodilian and mammal tracks, many very small and well preserved. Here we report diminutive, didactyl tracks (~1.0 cm long) assigned to a new dromaeosaurid ichnogenus *Dromaeosauriformipes*, which resembles the larger, but still quite small, ichnogenus *Dromaeosauripus*, also from the same formation only 30 km away. These diminutive tracks are consistent with the foot size of smaller dromaeosaurid taxa like Early Cretaceous *Microraptor* from China, and may represent diminutive species or juveniles of larger species. The association of tracks with lakeshore sediments is consistent with the evidence that *Microraptor* was a fish eater. Two trackways and isolated tracks indicate variable trackmaker gaits and speeds. If oviparous, as assumed for most non-avian dinosaur neonates, the trackmakers must have hatched from tiny eggs. Previous studies of the Korean Cretaceous indicate the presence of other diminutive (~1.0 cm long) theropod tracks (*Minisauripus*). Such occurrences strongly suggest that small tracks attributed to juveniles, or very small tetrapod species, are more common than previously supposed especially where suitable preservation conditions prevailed.

## Introduction

**Figure 1:**
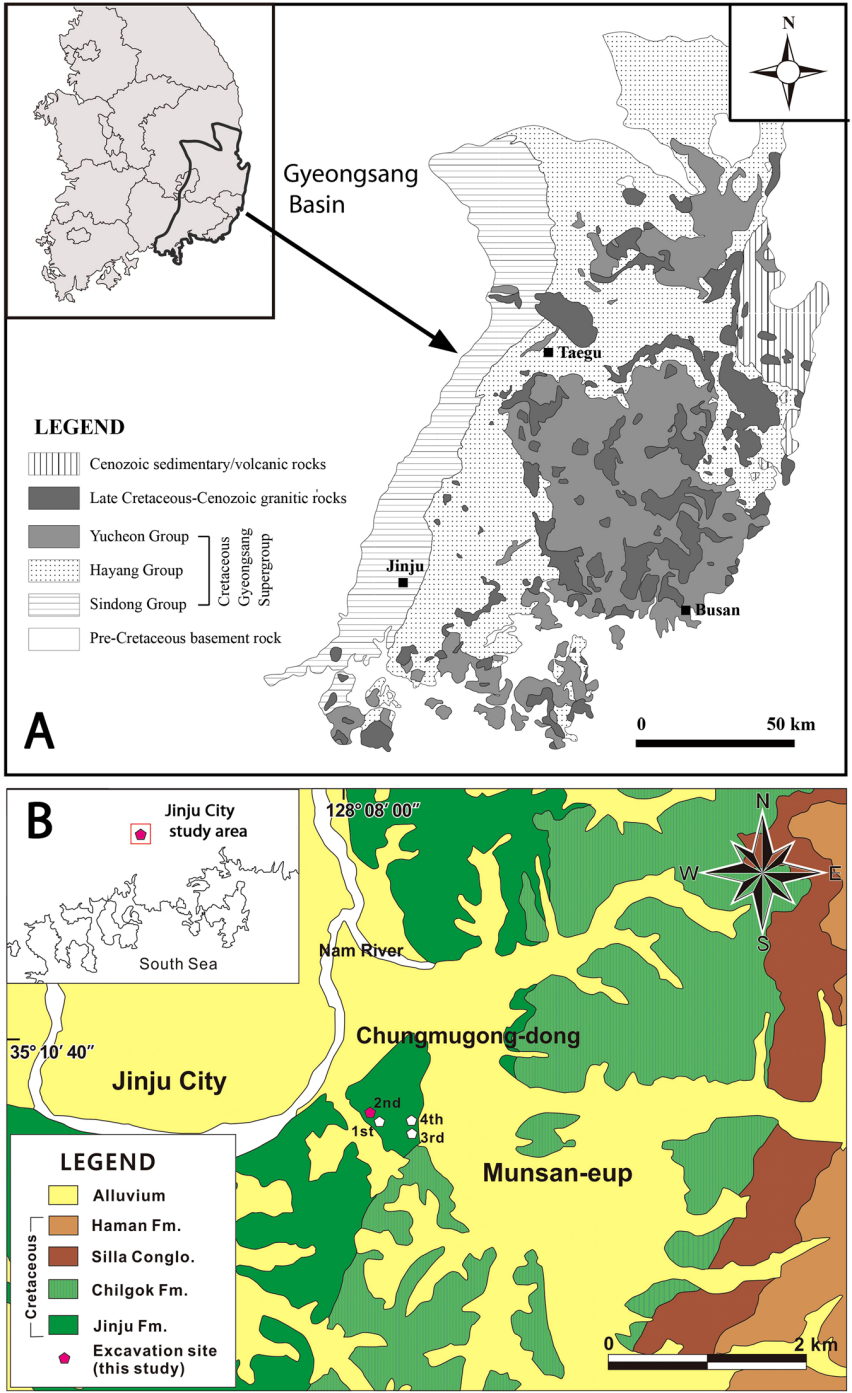
Study area locality map. (**A**) Group level general geology of Gyeongsang Basin in southeast of Korean peninsula (inset). (**B**) Formation level color map of geology of Jinju City area showing study area (inset) and detail of location of four excavation areas in the  Jinju Formation. Maps made by K-S K and M G L in Adobe photoshop (version CS6 www.adobe.com/Photoshop) and Canvas X (version, 2017 Build 160, http://www.canvasgfx.com/).

South Korea has become well known for the extraordinary abundance of Cretaceous vertebrate tracksites from the Gyeongsang Supergroup^1^, including the “Neocomian” Sindong Group and the Albian-Cenomanian Hayang Group^2^. It is beyond the scope of this short communication to review the extensive literature on these Cretaceous tetrapod tracksites. However, as essential context we draw attention to recent reports of abundant track discoveries in the Jinju Formation (Sindong Group) that include new mammal tracks^3^ and a suite of theropod tracks^4,5^ originating from one of four massive excavations and collecting projects at Jinju City (Fig. 1). The tracks originating from these excavations are abundant, diverse and well preserved, often representing small species that did not normally register tracks on less receptive substrates. Such superior track registration conditions which pertained during the deposition of the Jinju Formation resulted in the preservation of exceptional ichnofaunas that qualify for the label Konservat-Lagerstätte, simply defined as a site exhibiting extraordinary fossil preservation (Suppl. Info).

The region is already well known as the source of some of the most distinctive didactyl tracks attributed to dromaeosaurs and assigned to ichnogenus *Dromaeosauripus* from the Jinju^6^ and other formations^7,8^. Continuing the documentation of the Jinju project we here describe the occurrence of 18 diminutive didactyl tracks which we attribute to juvenile or diminutive dromaeosaur species and formally describe as *Dromaeosauriformipes rarus* ichnogen. et ichnosp. nov.

The remarkable abundance of Cretaceous tracks being discovered in Korea^6–8^ and China^9–14^, and the number of new ichnotaxa attributable to so called functional-didactyl ‘raptors’ (inferred dromaeosaurs)^6–14^ raises two intriguing, but challenging questions which we attempt to address below: 1) what is the size range of raptor tracks based on footprints or inferred from skeletal remains? and 2) how might diminutive tracks of juveniles be distinguished from those of adults?

## Methods

### Material recovery and documentation

**Figure 2:**
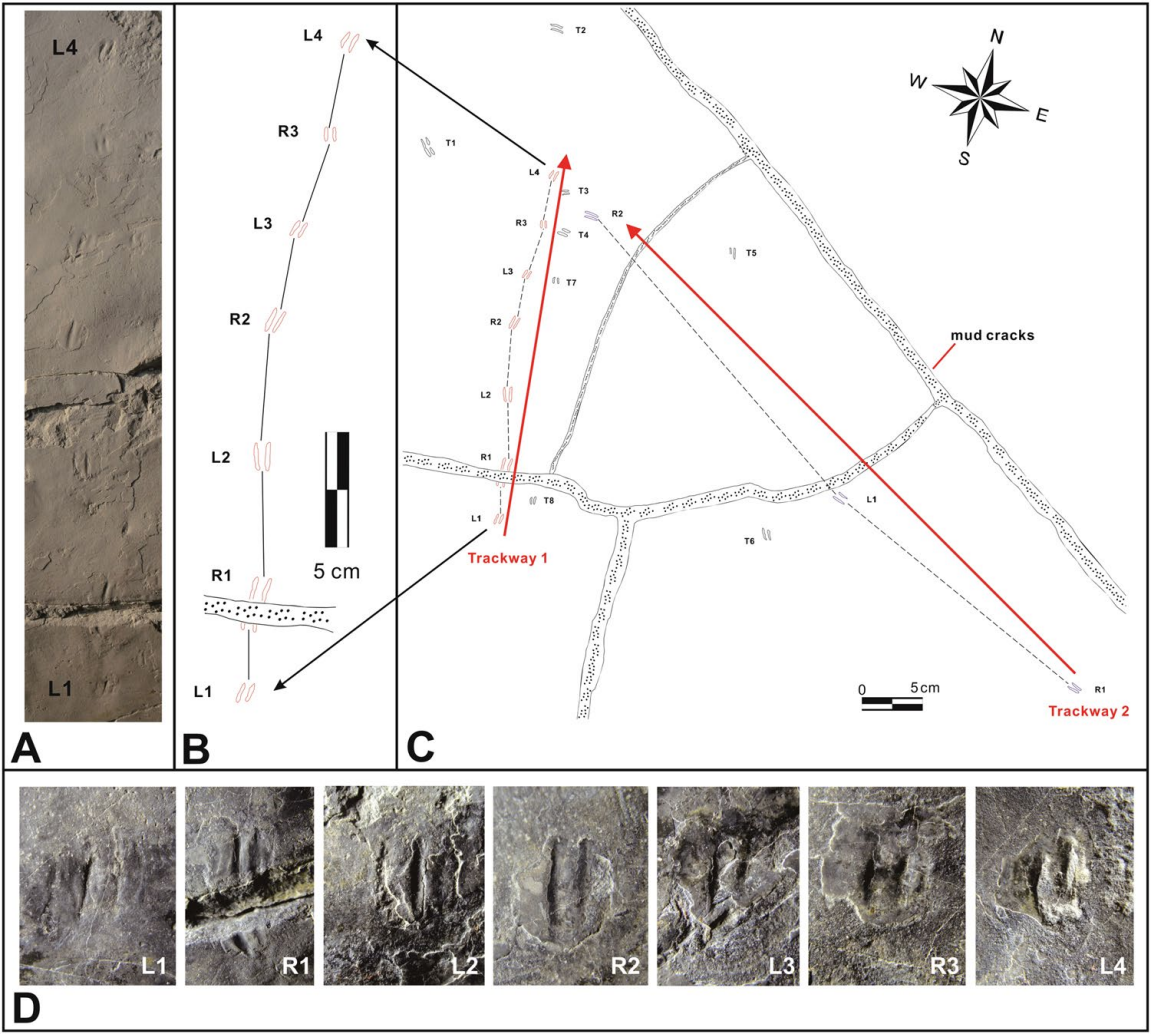
(**A**) Trackway 1 of diminutive dromaeosaurian (*Dromaeosauriformipes rarus* ichnogen. et ichnosp. nov.) consisting of seven footprints with short step. Original photo by K-S Kim. (**B**) Trackway 1 shown at same scale. (**C**) Track-bearing surface showing both Trackways 1 and 2, at half scale of A and B. (**D**) Individual tracks of trackway 1. Note the long step of the Trackway 2 trackmaker. Larger non- dromaeosaurian tracks also occur on this surface nearby. Drawing created with CorelDRAW X8 Graphic, version 18.1.0.661, https://www.coreldraw.com.

**Figure 3:**
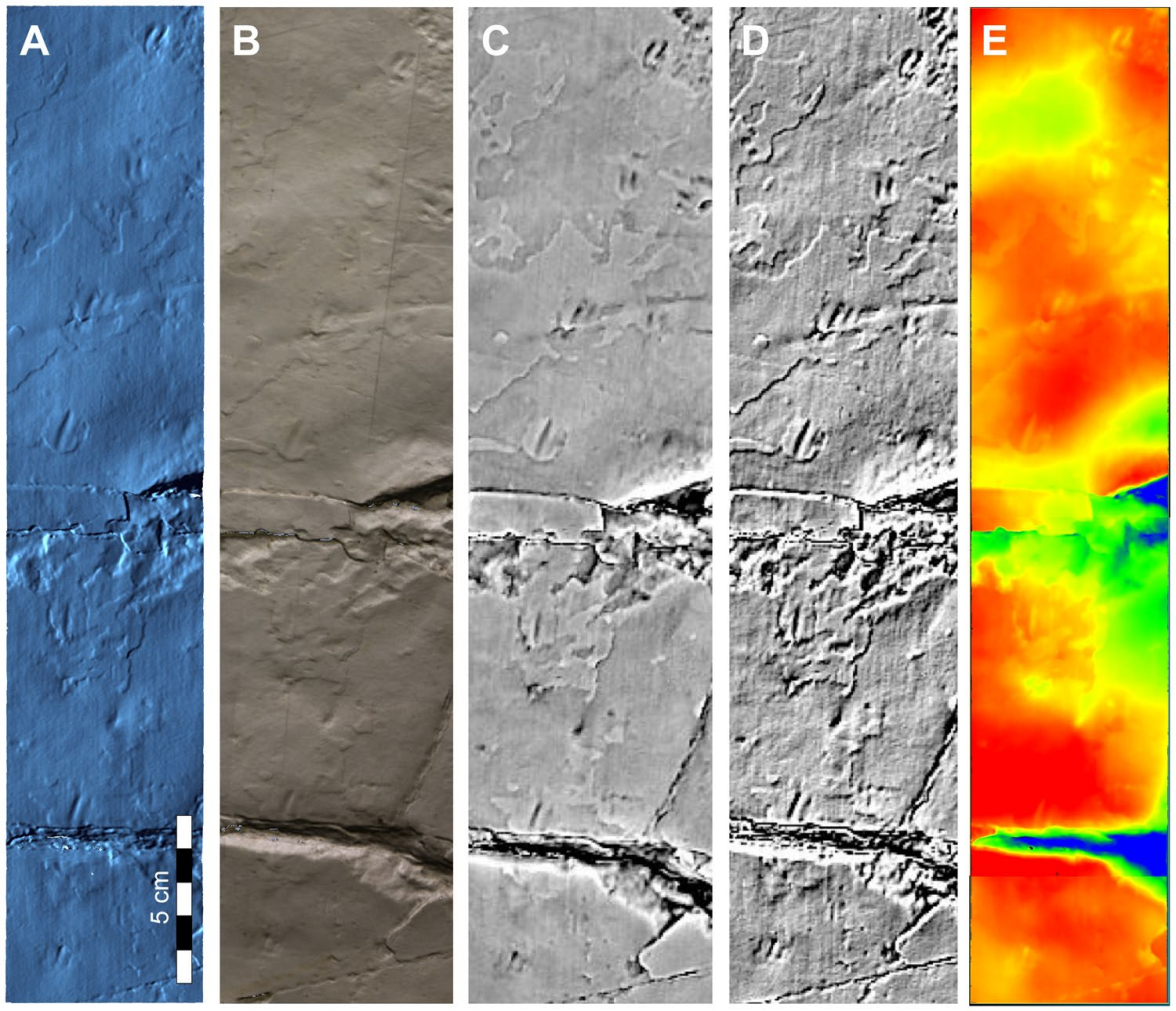
Images generated from three-dimensional models of trackway 1. (**A**) Mesh. (**B**) Point cloud with color. (**C**) Mean curvature shading. (**D**) Exaggerated shading. (**E**) Color depth. Modeling by J-H Ahn. See Supplementary Information for further details of methods.

**Figure 4:**
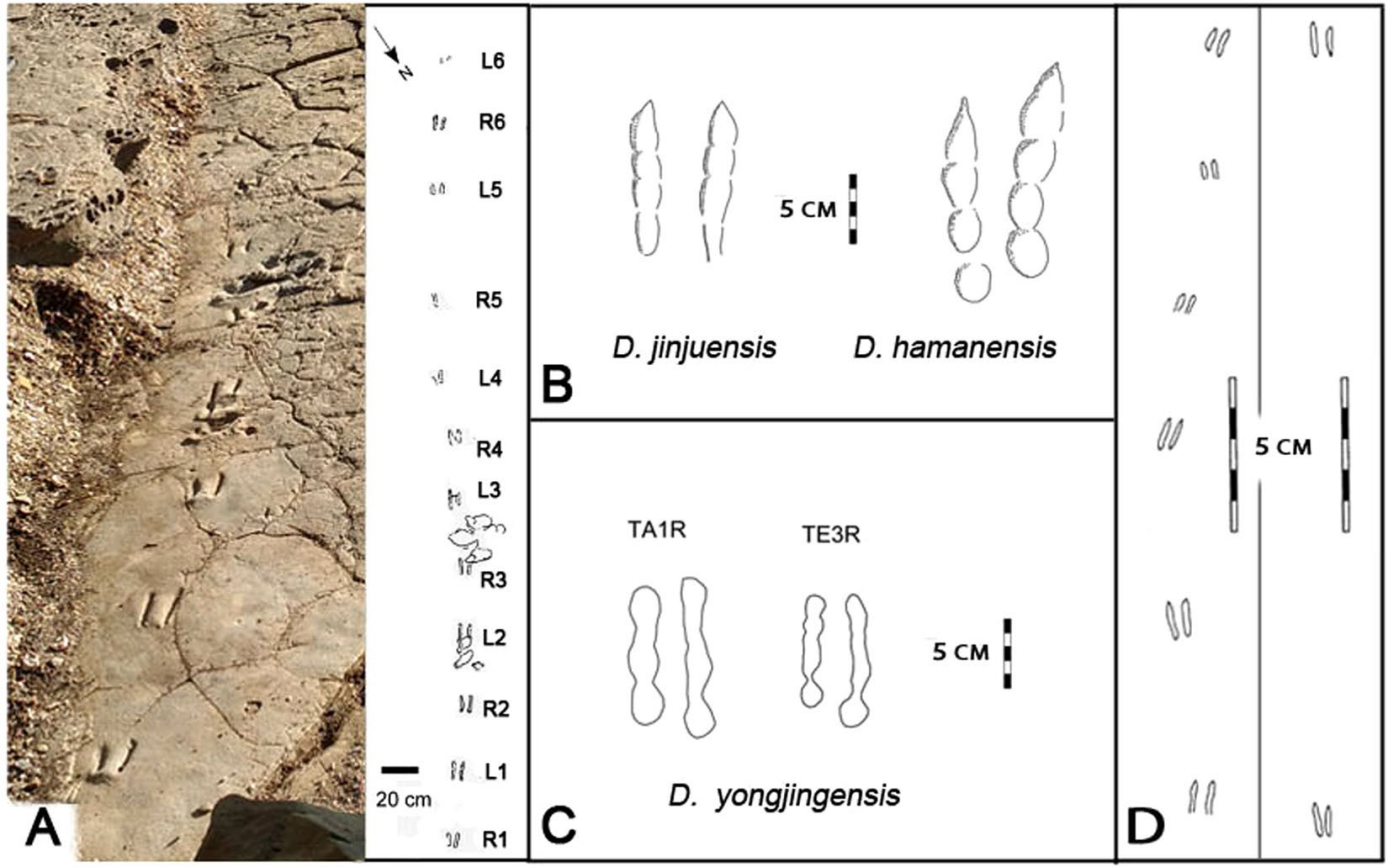
(**A**) The type trackway of *Dromaeosauripus jinjuensis* shown as photograph (left) and line drawing (right): (**B**,**C**) line drawings of single tracks from the type trackways of *D*. *jinjuensis*, *D*. *hamanesis* and *D*. *yongjingensis*. TA1R and TE3R identify individual tracks in *D*. *yongjingensis* sample^10^; (**D**) line drawings of the trackways of small *Dromaeosauriformipes rarus* ichnogen. et ichnosp nov. described here (compare with Figs 2 and 3). Photograph and line drawings by M. Lockley compiled in Adobe photoshop (version CS6 www.adobe.com/Photoshop).

As described elsewhere^4,5^ and in Supplementary Information, the excavations of track-rich Jinju Formation beds at Jinju City formed part of a Government mandated rescue mission around Korea National Natural Monument Number 534, named *The Pterosaur*, *Bird and Dinosaur Tracksite of Hotan-dong*, *Jinju* (Supplementary Information 1). Among the rescued specimens, cut out with large rock saw equipment^4,5^, is Chinju National University of Education (CUE) specimen CUE JI-2E Dr001 (Fig. 2), a slab containing two trackways of a diminutive didactyl biped and additional isolated tracks. All these footprints are approximately 1.0 cm long (Table 1). The tracks interpreted as continuous trackway segments are designated Trackways 1 and 2 with seven and three tracks respectively (Figs 2–4). There are at least eight other isolated tracks labelled as T1-T8. The surface also reveals two tridactyl tracks with quite different morphologies. One consisting of three parallel scratch marks up to 5 cm long, the other a large, more robust tridactyl track 16.2 cm long and 12 cm wide (SI Fig. SI3).

**Table 1:** Measurements of didactyl tracks/trackways from the Jinju Formation in Jinju Innovation City (National Natural Monument No. 534). Speed estimates calculated using the formula of Alexander^20^.

Trackway No.	Track No(n).	Track length (mm)	Track width (mm)	III width (mm)	IV width (mm)	Pace (cm)	Pace angle	Stride (cm)	Speed (m/s)
Trackway I	L1	9.40	3.84	1.94	1.44				
	R1	14.18	5.22	1.63	2.05	3.00	187.3		
	L2	12.74	5.12	2.03	1.82	6.11	182.2	10.05	
	R2	11.15	4.06	1.76	1.41	6.03	196.7	13.01	
	L3	7.91	3.43	1.60	1.93	4.24	189.1	10.26	
	R4	6.44	3.55	1.70	1.59	4.40	189	8.61	
	L4	9.54	3.91	1.69	1.68	3.94		8.41	
	**mean**	**10.33**	**4.15**	**1.76**	**1.7**	**4.62**	**188**	**10.00**	**0.6**
Trackway II	**R1**	11.29	2.78	1.83	1.94				
	**L1**	10.95	5.62	1.28	1.59	25.16	168		
	**R2**	9.33	3.85	1.69	1.81	31.28		55.63	
	**mean**	**10.5**	**4**	**1.6**	**1.77**	**28**			**10.5**
Isolated Tracks	1	17.27	3.70	1.30	1.53				
	2	10.70	3.19	1.83	1.97				
	3	6.91	2.32	1.11	1.27				
	4	8.82	3.77	1.45	1.55				
	5	9.70	4.0	1.27	1.37				
	6	10.89	3.24	1.37	1.45				
	7	5.44	3.68	1.15	1.22				
	8	6.49	2.87	2.15	1.43				
	**mean**	**9.53**	**3.35**	**1.45**	**1.47**				
	**Total mean**	**10.47**	**3.91**	**1.64**	**1.69**

All tracks were mapped and photographed *in situ*^4,5^ and in many cases replicated before the original specimens were excavated. In the case of CUE JI-2E Dr001 a replica was made of Trackway 1 which has been donated to the University of Colorado Museum of Natural History (UCM 214.309) for analysis as part of this study. The length (L), width (W) and steps (St) and strides (Str) between individual tracks were measured (Table 1) using the original specimens, replicas, tracings and calipers calibrated to the nearest 0.1 mm (Table 1). These measurements are shown graphically in Supplementary Information and Table 1.

## Results

### Description of tracks and trackways

The holotype trackway of *Dromaeosauriformipes rarus* ichnogen. et ichnosp. nov (Trackway 1), described below (Figs 2–4), consists of seven consecutive didactyl tracks averaging only 1.03 cm long and 0.41 cm wide (L/W 2.49) with and average step of 4.62 cm. Thus average step length is ~4.5 x footprint length. The mean pace angulation is 188°. Trackway 2 is very different in configuration consisting of three footprints (R-L-R) separated by relatively long steps. The tracks are similar in size to Trackway 1, averaging 1.05 cm long and 0.40 cm wide (L/W 2.63), but the two steps 25.16 and 31.28 cm (stride 55.63 cm) are about 5.5–6 times as long as in Trackway 1.

The eight isolated footprints that do not belong to Trackways 1 and 2 are numbered T1-T8. They are similar in size to the individual footprints in Trackways 1 and 2 but not obviously mutually associated with one another in trackways, although footprints T5-T6 and T7-T8 might represent steps of ~23.0 and 18 cm in two other trackways. Thus, the 18 tracks collectively represent an estimated 6 to 10 individual trackways possibly made by a similar number of different individuals. This inference is based on the convention that it is more parsimonious to count one individual per trackway (not per track)^15^, especially where footprint size varies, than to assume one individual making repeat crossings of a small area, at different speeds and in as many as ten different directions. The similarity in track depths at the Jinju site also suggests activity over a short period of time, rather than repeat crossings of the area by few individuals over a longer duration.

## Systematic Ichnology

### Systematic notes

The naming of tetrapod ichnotaxa at the ichnogenus and ichnospecies level is based primarily on morphology of the footprints, and trackway configurations, which in turn are influenced to various degrees by biological factors such as trackmaker foot morphology, size and behavior, but also by geological (physical environment) factors such as substrate conditions. There are well established conventions and guidelines that govern the introduction of new ichnotaxonomic names^16^ and aim to constrain variable or subjective interpretations. For example, as discussed below (Supplementary Information), differences used in naming new ichnotaxa at the ichnogenus or ichnospecies level may result, respectively, in a more conservative ‘lumping’ or a more liberal ‘splitting” approach.

Tracks of diminutive *Dromaeosauriformipes rarus* ichnogen. et ichnosp. nov., described here, are clearly distinct from all representatives of the much larger, previously-named ichnogenera (*Velociraptorichnus*, *Dromaeopodus*, *Dromaeosauripus*, *Menglongipus*, *and Sarmientichus*) attributed to functionally didactyl dromaeosaurid theropods^6,7,9–14^. However, it is morphologically more similar to *Dromaeosauripus*, represented by three ichnospecies, than to any representatives of the four other ichnogenera. *D*. *rarus* differs from the first-named *Dromaeosauripus* ichnospecies (*D*. *hamanensis*)^7^ in size, shape and digital pad configuration, and differs from *D*. *jinjuensis*^6^ from the Jinju Formation which is almost nine (8.96) times larger than *D*. *rarus* and much less elongate. However, the footprint to step length ratio between diminutive *D*. *rarus* and large *D*. *jinjuensis* is very similar. The choice of the ichnogenus label *Dromaeosauriformipes* recognizes both the similarities, in trackway proportions, and the differences, in footprint shape and size, between these two ichnotaxa.

### Systematic description

#### Order Reptilia

Dromaeosauriformipes ichnogen. nov.

**Etymology:** Ichnogenus named for similarity in “form” (shape) to the didactyl raptor track *Dromaeosauripus*, previously defined as meaning “dromaeosaur foot.” Ichnospecies from Latin *rarus* meaning both small and infrequently found.

**Holotype:** Trackway 1 on Chinju National University of Education (CUE) specimen CUE JI-2E Dr001 (Fig. 2), also represented by University of Colorado Museum of Natural History (UCM) replica UCM 214.309.

**Type locality and horizon:** Jinju Formation, Lower Cretaceous (Aptian), Korea National Natural Monument Number 534, Jinju Innovation City, Gyeongsangnam-do Province, South Korea (Supplementary Information Fig. S1).

**Diagnosis:** Didactyl tracks of a diminutive biped more than twice as long as wide, with two equidimensional, elongate parallel traces. Trackway about as narrow as individual tracks, with step length highly variable. Differs from *Dromaeosauripus* in size and degree of footprint elongation, and in the absence of any trace of digit II characteristic of other dromaeosaurid ichnotaxa.

*Dromaeosauriformipes rarus* ichnogen. et ichnosp. nov.: **Etymology:** Ichnospecies name from Latin *rarus* meaning both small and infrequently found.

**Holotype**, **locality and horizon:** as for ichnogenus.

**Description:** The holotype slab contains 18 tracks of a diminutive biped, preserved as natural impressions (concave epireliefs). These include the holotype series designated as Trackway 1 (Fig. 2), containing a seven-track sequence, a second three-track, paratype series, Trackway 2, and eight isolated tracks. All are remarkably consistent in size. The holotype averages 10. 33 mm long (L) and 4.15 mm wide (W): L/W 2.49 (N = 7). These mean values are similar to those obtained from Trackway 2 (L 10.5 mm. W 4.00 mm, L/W 2.63, N = 3), for the isolated tracks (means: L 9.53 mm, W 3.35 mm, L/W 2.84, N = 8) and for the sample as a whole (means: L 10.47 mm, W 3.91 mm, L/W 2.68, N = 18): Table 1.

The dimensions of holotype Trackway 1 reveal a mean step and stride length of 4.62 cm and 10.00 mm, respectively, indicating a step about 4.5 times footprint length and slow progression (0.6 m/sec, or 2.16 km/hr). By contrast paratype Trackway 2 has a mean step or pace length (PL) of 27.82 cm (stride of 55.63/2) indicating steps about 26.49 times footprint length and much higher estimated speed of 10.5 m/sec, or 37.8 km/hr. However, both trackways are very narrow, with correspondingly high pace angulation values (168°–188°).

## Discussion

### The deinonychosaurid/dromaeosaurid track record

All previous reports of didactyl, deinonychosaurid (dromaeosaurid) tracks including several from Korea^6–14,17–19^ have dealt with footprints an order of magnitude larger than those described here. These reports include the report of *Dromaeosauripus jinjuensis*^6^, which reveals footprints which are morphologically similar to the tracks described here, except for a huge size disparity. *D*. *jinjuensis* footprints average 9.26 cm long and 6.75 cm wide, thus being and order of magnitude larger. They are also little more than half (55%) as elongate than *D*. *rarus* (L/W 1.37 v. 2.49). The same order of magnitude size differences applies to comparisons between *Dromaeosauriformipes rarus*, described here, and all three ichnospecies of *Dromaeosauripus* (*D*. *hamanensis*^7^, *D*. *jinjuensis*^6^ and *D*. *yongjingensis*^12^). The latter two ichnospecies registered quite similar tracks^18^, lacking the fusion of the proximal traces of digits III and IV in *D*. *jinjuensis*, and in some, but not all examples of *D*. *yongjingensis*^7,10^. It is important to note that in both *D*. *hamanensis* and *D*. *yongjingensis* the tracks are more elongate (L/W 2.00 and 2.27 respectively) than in *D*. *jinjuensis* (L/W 1.37) due to the registration of a posterior heel or metatarsophalangeal pad. However, because diminutive *D*. *rarus* does not show heel traces, the L/W ratio (2.49) is a minimal estimate and would be greater if a heel traces had registered. Thus, the L/W ratio comparison with *D*. *jinjuensis* is based on measuring the same parameters (only distal digit traces), and comparisons with *D*. *hamanensis* and *D*. *yongjingensis* are only possible if length of heel traces are subtracted. The lack of any trace of the proximal portion of digit II in *D*. *rarus* is an ichnotaxomomically-significant difference between this diminutive morphotype and the type specimens of all other dromaeosaurid ichnogenera.

The trackway proportions of *D*. *jinjuensis* are similar to Trackway 1, with an average step and stride of 40.09 and 80.40 cm respectively, thus indicating a step about 4 times footprint length (SI Fig. SI4A–C). The *D*. *jinjuensis* pace angulation averages 174.7° compared with 188° for Trackway 1 described here for *D*. *rarus* (Figs 3 and 4). Trackways 1 and 2 indicate the trackmakers were highly mobile, and capable of progression at variable speeds. As noted above, speed estimates for Trackway 1 indicate slow progression at ~0.6 m/s (2.16 km/hr). The speed estimates for Trackway 2 (~10.5 m/s = 37.8 km/h: Table 1)^20^, suggest a striking variation in locomotor range (gait) for this trackmaker. However, this is within the range of estimates for other dinosaurs, and is consistent with the maximum running speed estimates for a dinosaur such as adult *Velociraptor*^21^. Likewise *Minisauripus* from Korea and China also indicate a wide range of variation in step or pace length, relative to footprint length (PL/FL)^22–24^ and thus considerable variation in trackway-derived speed estimates. For example, PL/FL for *Minisauripus* ranges from 5.18 to 9.07, 9.52 to 11.12 and 7.73 to 15.00 respectively for three samples from China^9,23,24^ and 6.14 to 18.0 for samples from Korea^8,24^: see SI Table 1.

It is well-established that deinonychosaurid tracks vary in both size and shape^18^ giving rise to the naming of several ichnogenera. Among these, in order of naming, *Velociraptorichnus*^9^, *Dromaeopodus*^10^, and *Dromaeosauripus*^7^ are based on well preserved types associated with trackway segments, and tracks showing digital pad traces. All are from Lower Cretaceous type localities in Asia. Ichnogenus *Dromaeosauripus* is represented by three aforementioned ichnospecies^6,7,12^ and indeterminate ichnospecies from the Lower Cretaceous of North America^19^. *Menglongipus*, also from the basal Cretaceous of China^11^ is based on material with suboptimal preservation. The *Velociraptorichnus* ichnospecies *V*. *zhangi* is also from the Lower Cretaceous of China^13^. Recent work suggests that ichnogenus *Sarmientichnus* is an extramorphological *nomen dubium* representing a poorly-preserved didactyl dinosaur track of deinonychosaur affinity^14^.

**Figure 5:**
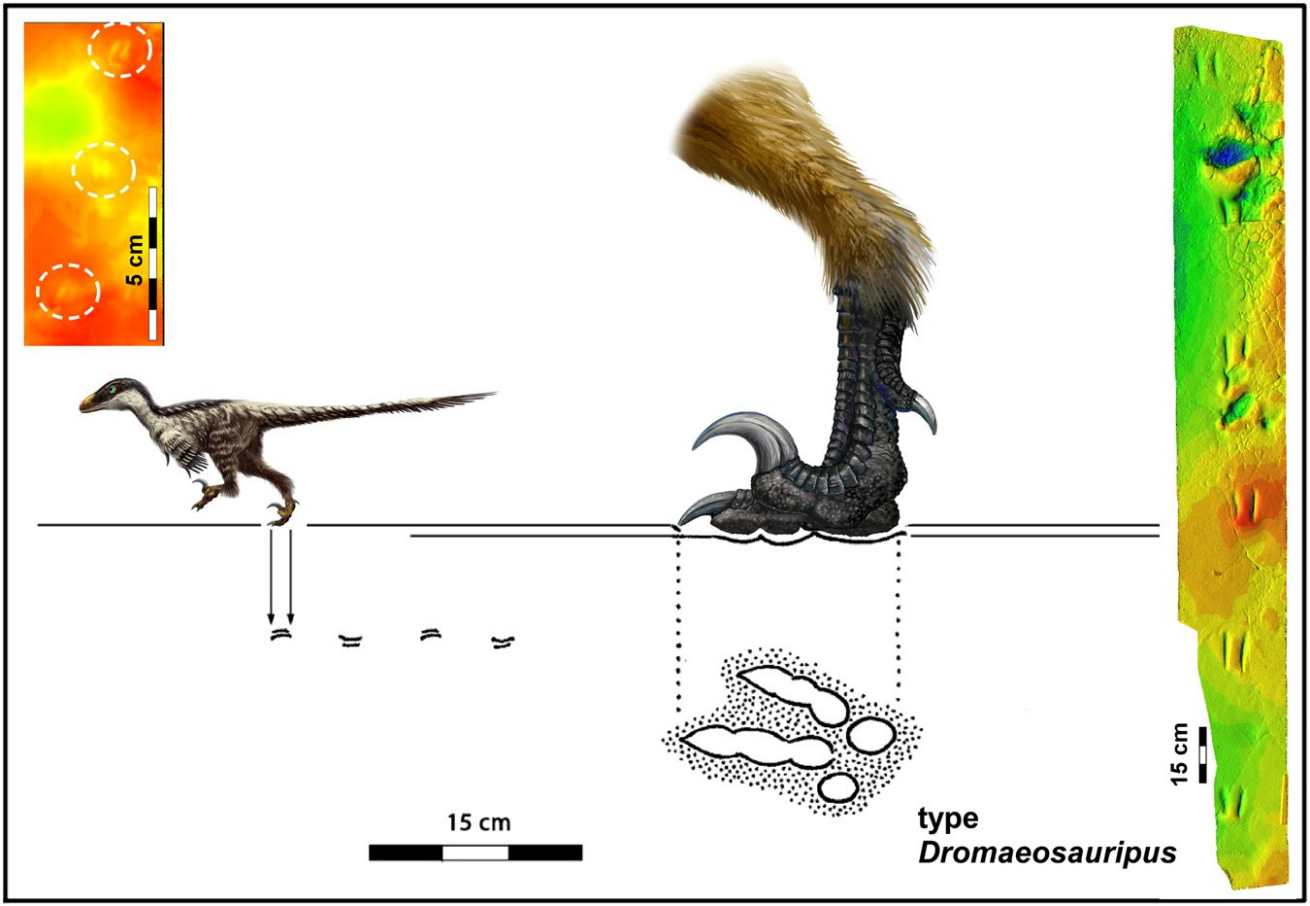
Illustrations of *Dromaeosauriformipes rarus* ichnogen. et ichnosp nov. trackway described here at same scale as type *Dromaeosauripus jinjuensis* with reconstruction of trackmaker anatomy at scale corresponding to footprint sizes. Upper left color inset is photogrammetric image of Trackway 1, and right side photogrammetric color image shows the type trackway of *Dromaeosauripus jinjuensis*^6^. Original illustration, artwork and 3D models compiled by MGL, XL, and K-S Kim in Photoshop (version CS6 www.adobe.com/Photoshop) and Canvas X (version, 2017 Build 160, http://www.canvasgfx.com/).

Previous studies indicate footprint lengths range between ~6.7 cm for *Menglongipus*^11^ and 28 cm for *Dromaeopodus*^10^. Thus the tracks described here (Figs 2–5 and SI4–5) are far smaller than any previously reported. Among named and well defined dromaeosaurid track morphotypes there are at least two preservational and/or behavioral morphotypes: 1) where the traces of digits III and IV converge and unite towards the heel, notably in *Velociraptorichnus* isp. and *Dromaeopodus* isp. and 2) those where separate toe traces are not joined at the heel, as in all examples of *Dromaeosauripus jinjuensis*^6^, diminutive *D*. *rarus*, and some *D*. *hamanensis*^7^ and *D*. *yongjingensis* trackways^12^. The variation in the large sample of trackways of *D*. *yongjingensis* suggests that the separate paired traces first reported for *D*. *jinjuensis* are a preservational or behavioral variant of deinonychosaurian tracks with digit traces which may be united in the heel^10–14^. If the lack of heel traces is due to a more digitigrade gait the explanation is behavioral, but if it is the result of substrate influences then the explanation is preservational. Both factors may be factors in any given cases (SI).

Based on trace morphology we conclude that at least 22 occurrences of deinonychosaurian tracksites, exhibiting traces of functionally didactyl bipeds were reported prior to the present discovery. Of these 14 were from China^9–14^, three from Korea^6–8^, three from North America^17,19^ and two from Europe^18^. The aforementioned differences in morphology, indicate that the diminutive tracks described here resemble *Dromaeosauripus* isp. in some features (separate traces of digits III and IV and proximal trace of digit II is not impressed)^6^. However, striking differences in size and elongation (L/W), and comparisons with other inferred dromaeosaurid tracks,^14,18,19^, marks *Dromaeosauriformipes rarus* as a new ichnotaxon distinct from any other existing ichnotaxon.

### Other potential trackmakers considered

It is important to consider the possibility of other potential, non-deinonychosaurid tracemakers. Based on the size of the individual traces, and their preservation as surface impressions (concave epireliefs) they are clearly surface trails. However, their small size suggests they could have been made by invertebrates. We have examined a number of surface trails attributed to invertebrates, mostly arthropods and illustrate them for comparison (SI Fig. SI4), showing both trackway configurations, and the morphology of individual tracks or traces that repeat within the trackways. These include the ichnogenera *Bifurculapes*, *Diplichnites*, *Hamipes*, *Kouphichnium*, and *Permichnium*^25^ While it is possible to see similarities between some of these individual traces (SI Fig. SI4) and those here labeled as small *Dromaeosauriformipes rarus* ichnogen. et ichnosp. nov., none of these ichnogenera have similar trackway configurations. In the case of the small didactyl traces described here, there is no evidence, other than size that they are of invertebrate origin. Moreover we are constrained by visible trace morphology to name traces according to ICZN conventions and guidelines^16^ and available literature^6,7,9–14,17–19^ (Suppl. Info). Thus, we infer, based on morphology, that the tracks were most likely made by a juvenile deinonychosaurid or a very small species representative of that clade. Again ICZN convention^16^ stresses that inferences about trackmaker identity are secondary to the description of track morphology.

### Interpreting the preservation and affinity of small trackmakers

Previous studies of the Korean Cretaceous have unequivocally established that preservation conditions in fine-grained deposits of the Sindong and Hayang groups, notably the Jinju, Haman and Jindong formations have provided excellent substrate conditions for the preservation of both small and large tetrapod tracks. These include the dinosaur track *Minisauripus*^8,24^, and the lizard track *Neosauroides*^26^ from the Haman Formation and the mammal track *Koreasaltipes* from the Jinju Formation^3^.

Currently *Koreasaltipes*, is the smallest fossil mammal track known, Korean *Minisauripus*, which also occurs in China, is the smallest representative of this ichnogenus, and the *Dromaeosauriformipes rarus*, described here, is clearly the smallest known didactyl track inferred to represent the dromaeosaurid clade. As there is no paleoecological evidence to suggest that Korea was home to exceptionally small tetrapods, the reason for these occurrences probably pertain to exceptional, preservation conditions. Such positive biases favoring preservation of small tracks have important paleontological implications. The significance of small tracks and the biases against their preservation have been discussed by several authors, often inconclusively^8,23,26–29^. Possible and partial interpretations for lack of small tracks are as follows: 1) in comparison with large tracks, small tracks are rarely preserved, 2) trackmakers grew rapidly, perhaps not leaving their nests until having reached relatively large sizes, 3) small species were rare, 4) before burial and/or after present day exhumation and exposure to erosion, small tracks are easily eroded, 5) it is not easy to find smaller tracks due to observer oversight.

The first argument is compelling given that many substrates are unsuitable for preserving tracks of small, lightweight trackmakers. This argument is also supported by the concentration of small tracks in certain optimal preservation facies (Konservat-Lagerstätte type), such as the lake basin settings dealt with here.

The second argument pertaining to trackmaker growth most likely applies to relatively large species, and depends on finding evidence of a range of track sizes pertaining to particular ichnotaxa. For example, the Korean Cretaceous has produced a relatively full range of sauropod track sizes^30^. There are also differences, for example, between birds (avian-theropods) which are precocial and leave the nest early with track making capability when juvenile and small, and altricial species which may be relatively large by the time they leave the nest.

The third argument, that small species were rare, although refuted by the skeletal record, raises the question of whether small tracks represent small species or juveniles of larger species. Differentiating between such interpretations depends, partly, on the range of size of tracks of a particular morphotype (ichnotaxon). For example, it has been inferred^8^ that *Minisauripus* represents a small species because there are, in a global sample of about 100 tracks, no convincing examples of tracks longer than between ~1.0–~6.0 cm: hence little evidence of large adults. Based on the tentative inference that two large tracks (16.1–20.0 cm long) might possibly represent adults of the *Minisauripus* morphotype^8^ the alternate possibility that small *Minisauripus* represented juveniles was noted^8^, but not strongly supported by later finds^23^. The question of juveniles, vs. a small species remains open. However, in the case of the *Dromaeosauriformipes rarus* tracks described here, we have good evidence of large tracks, *Dromaeosauripus jinjuensis* (Fig. 5), from the same formation, albeit at only one site 30 km away. Thus, we could either infer that the tracks represent juveniles, or a very small species. There is no evidence to reject the latter possibility of multiple, co-existing species. On the contrary, there is growing evidence that a large number of Asian dromaeosaurid (microraptorine) species were small^31–33^. These include two species of *Microraptor* from China^31–33^, the smaller of which (*M*. *zhaoianus*) had feet only ~2.5 cm long, based on total length of digit III phalanges. These measurements are much closer to the *D*. *rarus* tracks described here than to any of the larger tracks (Figs 2–5, SI Figs SI4 and 5). There are also very small North American species including *Hesperorhynchus*^34^ and *Bambiraptor*^35^, the former of which has been estimated to have a body weight of only 1900 grams (~2.0 kg). Evidence that *Microraptor* was a fish eater^33^ provides support for the proposed microraptorine origin of the *D*. *rarus* tracks described here, since they were found in a lakeshore setting.

The inference that small tracks are rare^27–29^ has been increasingly challenged by more finds and ongoing studies of the Korean Cretaceous where small tracks of avian theropods (birds), non-avian theropods, pterosaurs, lizards and mammals are abundant. The growing database offers the opportunity consider the ontogeny of various trackmaking groups, and such paleobiological questions as hatchling or birth size and related questions of oviparity and viviparity. However, the data base is still small and it is recognized that without compelling evidence that juveniles and adults were active together, such as in social gregarious groups^29^ the occurrence of *Dromaeosauripus jinjuensis*^6^ and *Dromaeosaurifromipes rarus*, described here, in the same formation, but 30 km apart, is not an association from which unequivocal biological or ontogenetic inferences can be drawn. We can only infer that presumed dromaeosaur track makers, much smaller than the maker of *D*. *jinjuensis* tracks, and with more elongate feet, were active at about the same time, and that Chinese discoveries confirm that small microraptorines^31–33^ are known in the greater region (SI Figs 5 and 6). Given reports that small theropod tracks tend to be narrow, or elongate, whereas large tracks tend to be wide (Suppl. Info), there is precedent for inferring that the feet of dromaeoasurid trackmakers also grew wider during ontogeny.

Many extant squamates (lizards and snakes) are viviparous, but all “other extant Reptilia (turtles, crocodilians, spenodontids, and birds) are entirely oviparous^36^.” It is therefore assumed that non avian dinosaurs were also oviparous^36^. It has been suggested that some sauropsid dinosaurs were also oviparous^37,38^, but given the abundant evidence for eggs and nests, this speculation has not been well received^36^. Thus, the weight of evidence does little to support speculations of dinosaur viviparity. However, most known dinosaur eggs are relatively large (>10 cm diameter), and in many cases produced hatchlings that would have produced trackmakers larger than those inferred here. However, in the case of *Minsauripus*^8.23,24^ there is unequivocal evidence that some such small trackmakers registered tracks only ~1.0 cm long. *Minisauripus* tracks are abundant in the Haman Formation but were also recently discovered, as exceptionally well-preserved specimens, in the Jinju Formation (Suppl. Info), again supporting the wealth of evidence for diminutive trackmakers. Such small tracks (L: 1.0–2.5 cm) suggest diminutive trackmakers that likely hatched from smaller eggs. Alternatively, one might infer they belonged to small species that gave live birth. If altricial, with a period of growth in the nest, as in the case of many small extant birds (avian theropods) like passerines, the eggs may have been as small as those laid by small sparrow-sized birds^8^. If the species was precocial, as in the case of many extant shorebirds (charadriformes) the eggs may have been larger, with size presumably depending on the size of the adult egg layer^38,39^.

Despite these considerations which suggest oviparity throughout the Dinosauria recent evidence from the study of *Dinocephalosaurus* (an archosauromorph) suggests “no fundamental reason archosauromorphs could not achieve live birth”^36^. The evidence that some early archosauromorphs were viviparous does little to support or refute the idea that diminutive theropod tracks might represent viviparous species. Likewise, given that most extinct viviparous reptiles, including *Dinocephalosaurus*, were marine adapted^40^ there is no compelling support for viviparity among small terrestrial theropods. Thus, on balance, *D*. *rarus* tracks described here are attributed to small hatchlings of a species of unknown adult size, but consistent with several of the smaller microraptorine dromaeosaurid dinosaurs known from the Asian fossil record.

## Electronic supplementary material

Supplementary Information
