# Supplementary material for: Smallest known raptor tracks suggest microraptorine activity in lakeshore setting
Source: Sci Rep. 2018 Nov 15;8:16908. doi: 10.1038/s41598-018-35289-4 (PMC6237872; doi:10.1038/s41598-018-35289-4)
Supplement: Supplementary file 1 — Supplementary Information [file 41598_2018_35289_MOESM1_ESM.pdf]

Smallest known raptor tracks suggest microraptorine activity in lakeshore setting

Supplementary Information

Kyung Soo Kim[1], Jong Deock Lim[2], Martin G. Lockley[3], Lida Xing[4], Dong Hee Kim[5], Laura Piñuela[6], Anthony Romilio[7], Jae Sang Yoo[1], Jin Ho Kim[1], Jaehong Ahn[8]

[1] *Department of Science Education, Chinju National University of Education, 3 Jinnyangho-ro 369beon-gil, Jinju-si, Gyeongnam 52673, South Korea*

[2] *Cultural Heritage Administration, Government Complex-Daejeon, 18, Cheongsa-ro, Seo-gu, Daejon 35208, South Korea*

[3] *Dinosaur Trackers Research Group, University of Colorado Denver, P.O. Box 173364, Denver, CO 80217-3364, USA*

[4] *School of the Earth Sciences and Resources, China University of Geosciences, Beijing 100083, China*

[5] *National Science Museum, 481 Daedeok-daero, Yuseong-gu, Daejeon 34143, South Korea*

[6] *Museo del Jurásico de Asturias MUJA (Jurassic Museum of Asturias), Colunga E-33328, Spain*

[7] *School of Biological Sciences, the University of Queensland, Brisbane, Qld 4072, Australia*

[8] *Graduate School of Culture Technology, Korea Advanced Institute of Science and Technology, 291, Daehak-ro, Yuseong-gu, Daejeon 34141, South Korea*

N.B. references in superscript [(4-7, 19 & 26)] refer to main text. SI references 1-11 , which are not in main text, occur at end of Supplementary Information.

1 Excavation and documentation methods

As described in previous papers dealing with tetrapod tracks from the Jinju Innovation City excavations [main text refs [4,5]], large numbers of tracks and trackways were excavated from multiple track-bearing levels, at 4 excavation sites (1-4), at the site now designated as Korea National Natural Monument Number 534, [*The Pterosaur, Bird and Dinosaur Tracksite of Hotan-dong, Jinju*]. Wherever possible important specimens were removed to preserve unbroken trackway segments. For example, the extent of large the excavation area from which the specimen described here originated, is shown in Fig. SI 1, and the specimen itself is shown in Fig SI 2. As described and illustrated in the main text (Figs. 2-4) the specimen is curated and identified as Chinju National University of Education (CUE) specimen CUE JI-2E Dr001. The slab contains two trackways of a diminutive didactyl biped and additional isolated tracks of this type as well as two tracks of larger tridactyl trackmakers.

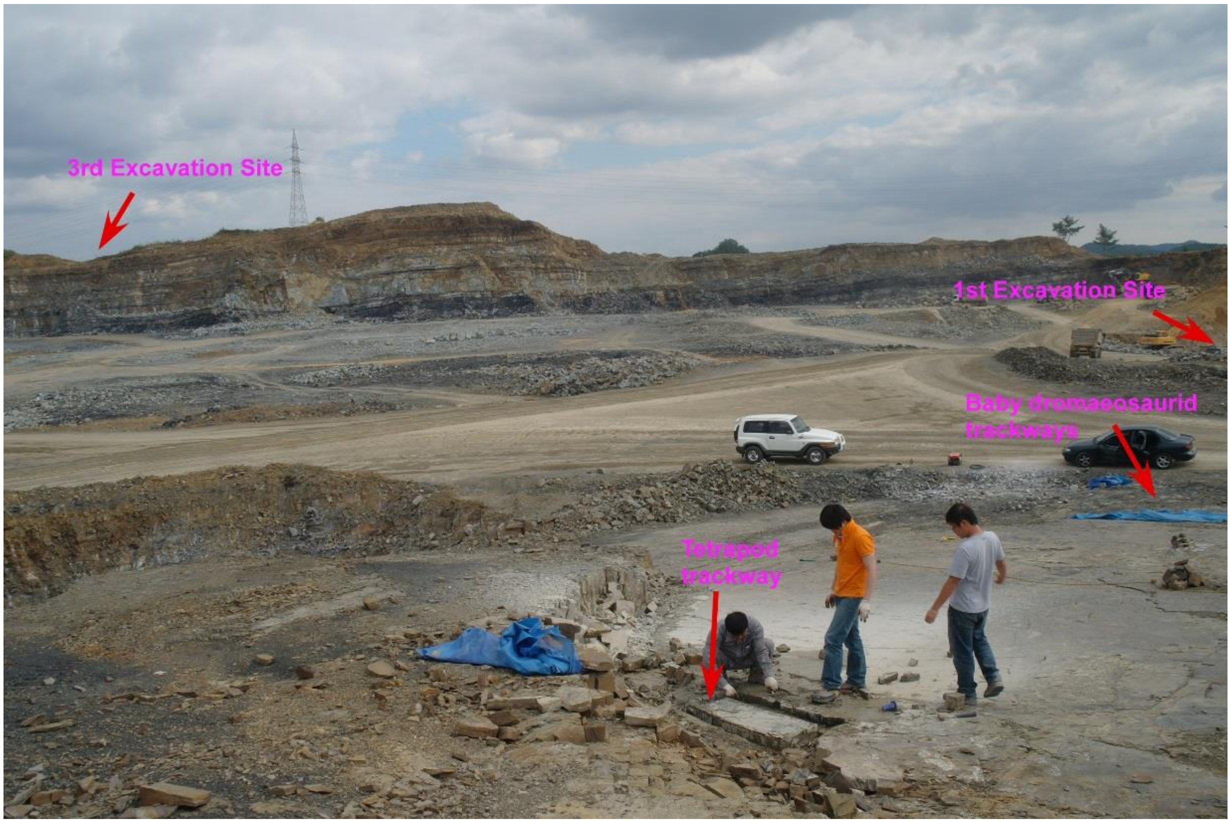

Fig. SI 1. Part of the excavated area at Jinju Innovation City (Korea National Natural Monument Number 534) showing the in situ source of the slab labelled “Baby dromaeosaurid trackways” containing tracks hereafter referred to as *Dromaeosauriformipes rarus* ichnogen. et ichnosp. nov. Photo by K S Kim

In all cases of trackway discovery and preparation of trackways for removal, specimens were photographed, replicated by silicon rubber, mapped and measured in place, to preserve the maximum of authentic data on size of tracks, trackway configurtion parameters (step, stride pace angulation, trackway width etc.,) and trackway orientations. In the case of Chinju National University of Education (CUE) specimen CUE JI-2E Dr001, where the focus of attention was on the small dromaeosaurid tracks, the position and size of associated larger tracks was recorded, including a tridactyl theropod track ~16.2 cm long and 12.0 cm wide and three parallel scratch marks (Fig. SI 3).

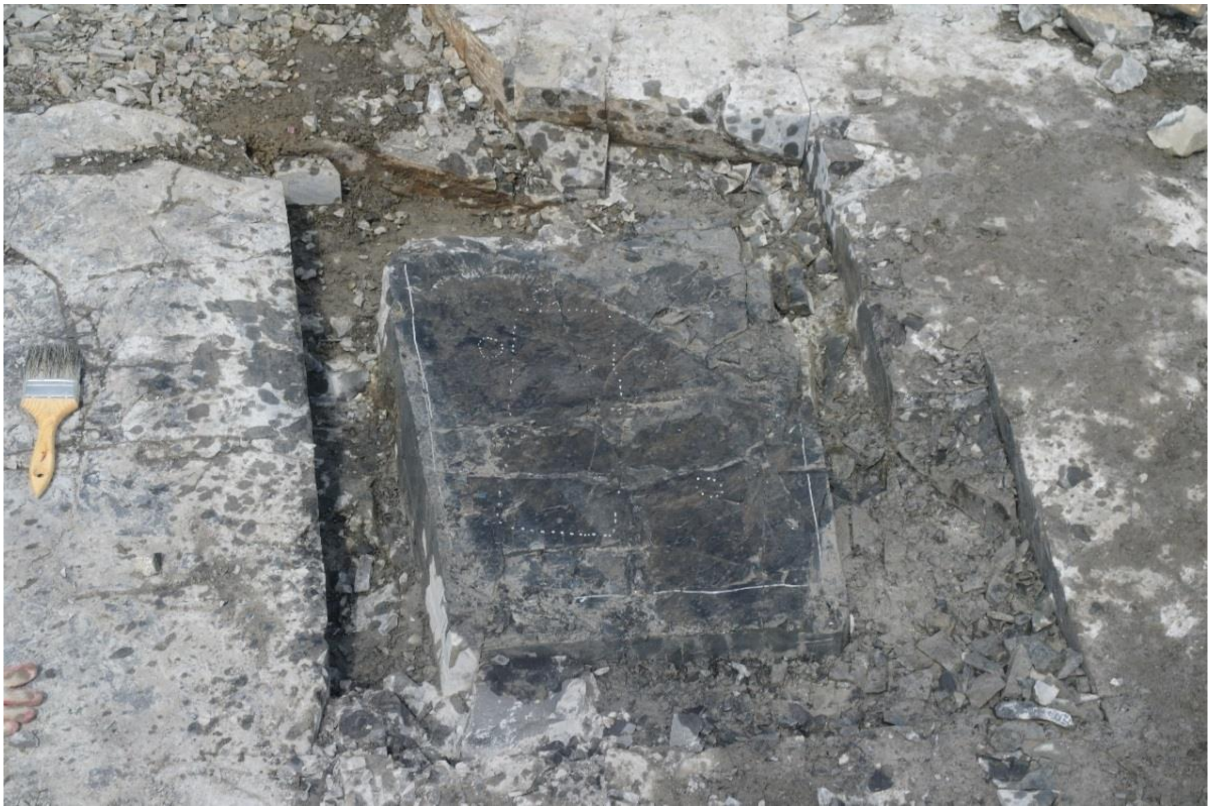

Fig SI 2. The “baby dromaeosaurid trackways” slab, the original location of which is shown in Fig SI 1 with tracks identified in field marked by outline of white dots. Compare with map in main text (Fig 2) and Fig SI 3. Photo by K.S Kim

Three-dimensional data for trackway 1 (main text Fig. 3) were acquired using laser scanning and photogrammetry techniques. The plaster specimen was scanned with Konica-Minolta Vivid 9i (a triangulation-based laser scanner with up to ± 0.010 mm accuracy). Point cloud data were processed and converted into a mesh model in Geomagic Design X (v. 5.1.0.0, http://www.3dsystems.com/)). The mesh model was rendered with non-photorealistic rendering like exaggerated shading and mean curvature shading in an in-house software for clear visualization of tracks (by Jaehong Ahn). A color map image was generated from the model to visualize depth information in Paraview (v. 2.6.1, open source software, http://www.paraview.org/)). These data are available as Supplementary Information files. For three-dimentional modeling of *Dromaeosauripus jinjuensis*, 119 photos were taken in situ with Nikon D600 (focal length 17-55 mm, 300×300 resolution) to build a photogrammetric model in Autodesk ReCap (v. 4.2.02, https://help.autodesk.com/view/RECAP/2018/ENU/)).

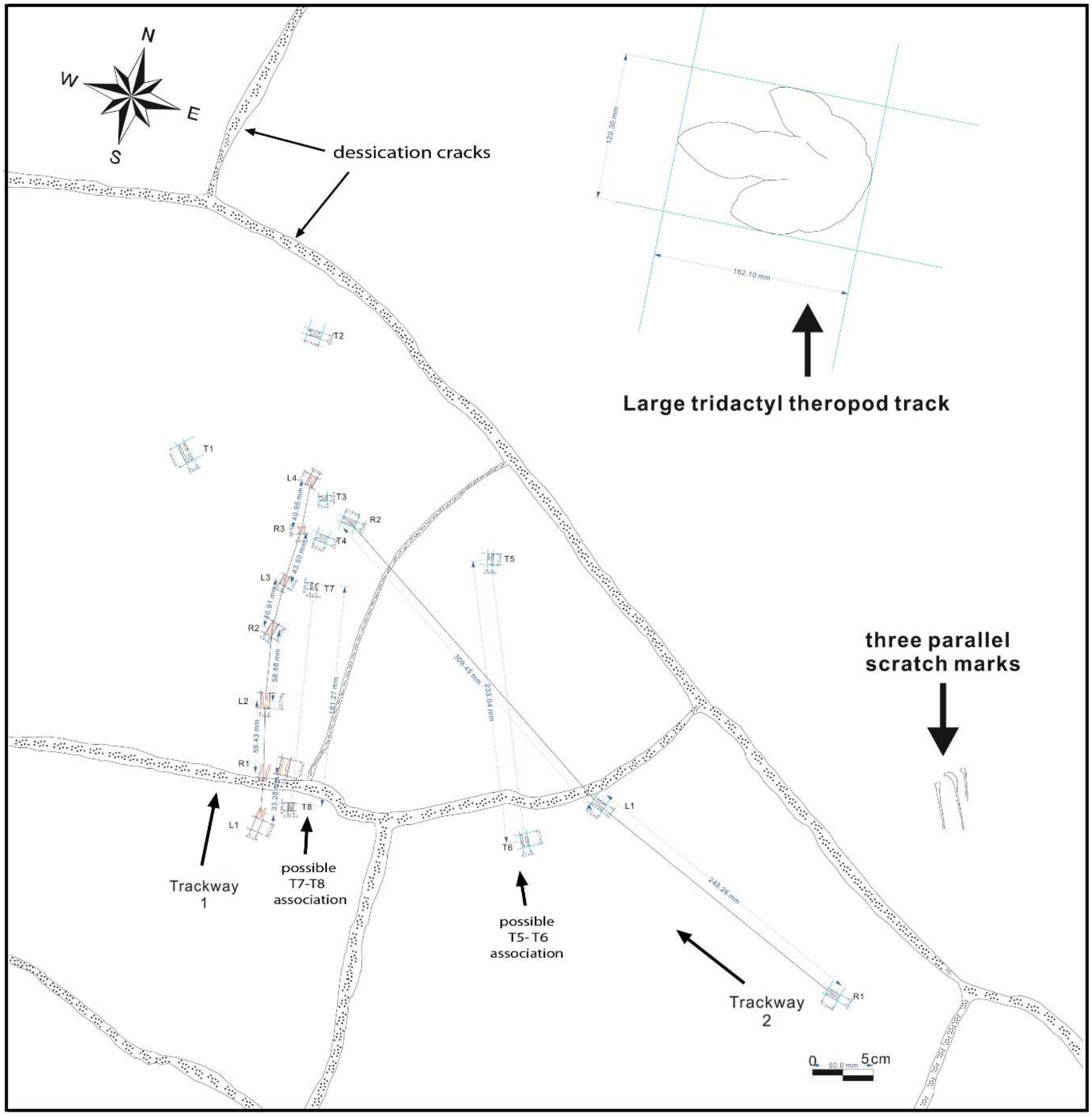

Fig. SI 3. Map of the 'Baby dromaeosaurid trackways' slab (Figs. SI 1 and SI 2) showing the presence of large tridactyl tracks, and measurements made in the field prior to trackway excavation. Note trackways 1 and 2 marked by dashed line and possible association of tracks T5-T6 and T7-T8 in trackways (dotted line). Maps made by K-S Kim, J.H. Kim and M G L in Adobe photoshop CS6 (www.adobe.com/Photoshop) and Canvas X (version, 2017 Build 160, http://www.canvasgfx.com/).

2. Comparison between *Dromaeosauriformipes* and invertebrate traces

As summarized in the main text, the small *Dromaeosauriformipes rarus* ichnogen. et ichnosp. nov., tracks, including Trackways 1 and 2, which show short and long steps respectively, are small enough to have been made by invertebrates. In order to evaluate the possible merit of such an interpretation we have compared the trackways with surface trails (trackways) of invertebrates, generally inferred to have been arthropod trackmakers, and especially with those that have any paired, individual trace sets (footprints) that might be confused with *Dromaeosauriformipes*, or lead to the conclusion that the trackmaker was not a small didactyl biped, as we infer. Our primary source of information on invertebrate trails has been the Treatise on Invertebrate Paleontology (part W) [main text ref [25]] the standard compendium on Trace Fossils. We have also refered to the primary references cited therein. The invertbrate traces showing any similarity to *Dromaeosauriformipes rarus* ichnogen. et ichnosp. nov. are shown in Fig. SI 4.

The only invertebrate traces with paired traces oriented more or less parallel to the trackway axes are *Bifurculapes* (Fig. SI 4E) and *Hamipes* (Fig. SI 4F). In both cases the trackways consist of a double row of traces, in contrast to the single narrow trackway (single row) of *Dromaeosauriformipes rarus* ichnogen. et ichnosp. nov., which show much longer spacing along the trackway axis (Figs.SI 4C-D). We use the ratio of step length / trackway width as a measure of trackway narrowness. The values for *Dromaeosauriformipes rarus* ichnogen. et ichnosp. nov. are approximately 5.0 in Trackway 1, and 25.0 in Trackway 2, indicating very narrow trackways and great vartiation in step length of long legged trackmaker. The values for *Dromaeosauripus hamanensis* and *Dromaeosauripus jinjuensis* are approximately 7.3 and 4.1, respectively (Figs. SI 4A-B) [main text refs [6-7]]. By contrast the values for *Bifurculapes* and *Hamipes* are approximately 0.5 and 0.35 respectively, indicating very wide trackways relative to short step lengths, which we infer to be the result of short legged trackmakers. We also note that the width of the traces in *Bifurculapes* and *Hamipes* are very thin in comparison with *Dromaeosauriformipes rarus* ichnogen. et ichnosp. nov., thus indicating very delicate appendages as seen in many arthropods.

The trackways of *Diplichnites* (Fig. SI 4G) and especially *Permichnium* (Fig SI 4H) also have what may appear to be "paired" traces, but they are rotated so as to bifurcate outwardly, or laterally, at a high or perpendicular angle to the trackway axis. The step length / trackway with ratios are also very low, approximately 0.1 and 0.3 respectively. Lastly the limulid trace *Kouphichnium* (Fig. SI 4I) studied in detail by Caster (1938)[SI ref 1] is known to vary in size and have many variable morphological

expressions, due to the limulid trackmaker having multiple appendages with variable morpholgy. However, none of the variable expressions of idealized *Kouphichnium* morpholopgy have been reported that remotely resemble the *Dromaeosauriformipes* trackways described here. Notably the step-length / trackway width ratio (~0.6) is also more typical of arthropods than any bipedal vertebrate. These comparison lead us to conclude that there are no known invertebrates that could be inferred to have made the surface trails here identified as the dromaeosaur trackways.

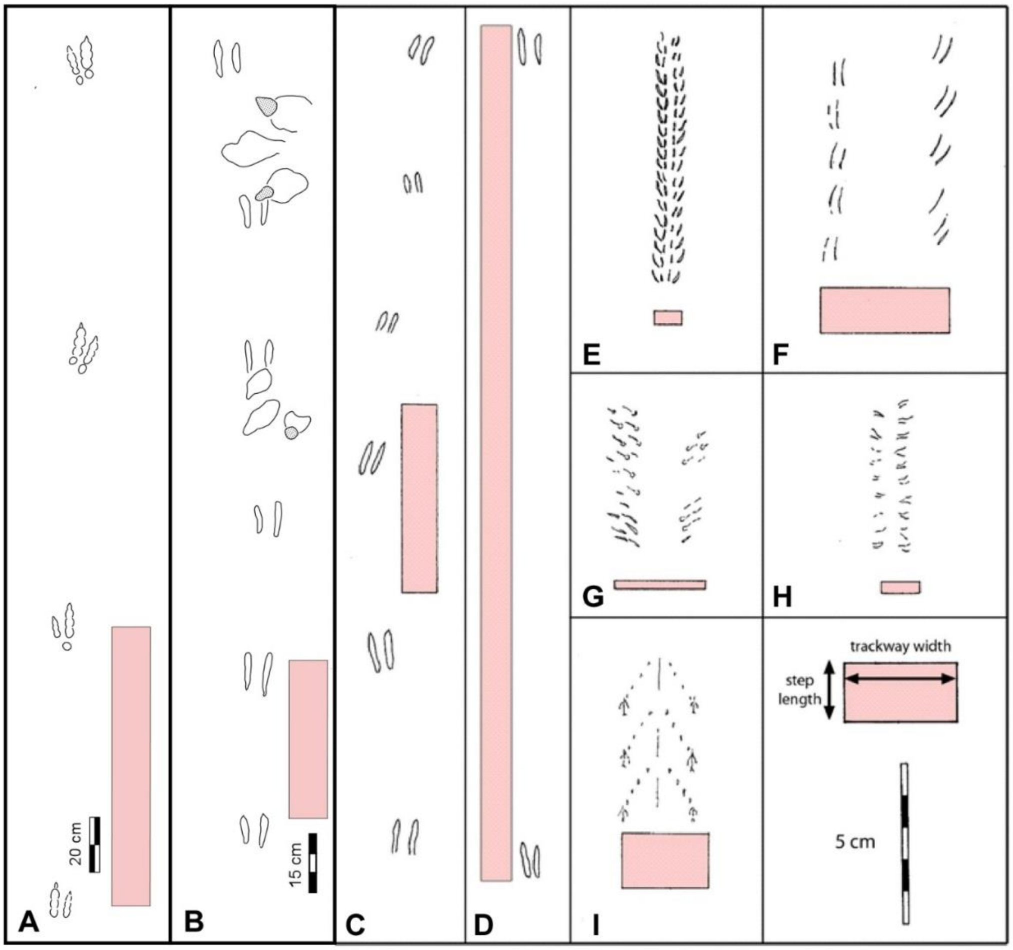

Fig SI 4. A-B : Large dromaeosaur trackways. C-D: Baby dromaeosaur trackways (see main text Figs. 2-4) compared with invertebrate traces (E-I) showing length of step and trackway width as a ratio (pink rectangles). C-I are same scale. A: *Dromaeosauripus hamanensis* trackway [main text ref [7]], B: *Dromaeosauripus jinjuensis* trackway [main text ref [6]], C: *Dromaeosauriformipes rarus* ichnogen. et ichnosp. nov. Trackway 1 with short step, D: *D. rarus* ichnogen. et ichnosp. nov. Trackway 2 with long step, E: *Bifurculapes*, F: *Hamipes*, G: *Diplichnites*, H: *Permichnium*, I: *Koupichnium*. E-I after Hantzschel[19]. Illustrations made by K-S K and M G L in Adobe photoshop CS6 www.adobe.com/Photoshop

3. Inferred trackmaker identification

The body fossil record of dromaeosaurid dinosaurs indicates that species diversity within the clade covers a wide range of body sizes from *Microraptor* [main text refs [30-31], SI ref. 2] through to *Utahraptor*-sized species that probably made the 28 cm long tracks reported from the Lower Createceous of Shandong, China [ref [12]]. As shown in Figures SI 5 and SI 6 the lengths of the digit III traces can be estimated with considerable accuracy from measurements of the total length of digit phalanges from articulated feet [33]. These give estimated footprint lengths (~2.5 cm) which are much closer to those represented by the diminutive *Dromaeosauriformipes rarus* ichnogen. et ichnosp. nov. tracks than to the tracks of three previoulsy named *Dromaeosauripus* ichnospecies which indicate foot lengths of between about 10.0 cm and 15.0 cm.

Recent reports of an inferred dromaeosaurid trackway from the Lower Cretaceous of Shaanxi Province, China (text ref [14]) indicates that under certain conditions of preservation dromaeosaurid may be registered as a single linear trace, as if the trackmaker were monodactyl as was inferred for the ichnogenus *Sarmientichnus*, originally reported from Argentina. This study[14] demonstrated that *Sarmientichnus* is an extramorphological *nomen dubium* (or form ichnogenus) demonstrably made by a didactyl biped (presumably a dromaeosaurid) in the case of the Shaanxi tracks, and not a useful or valid ichnogenus diagnostic of the trackmaker's foot morphology. By contrast *D. rarus* is morphologically similar to several ichnospecies of *Dromaeosauripus*, and is inferred to reflect trackmaker foot morphology. The ichnogenus name *Dromaeosauriformipes* was deliberately chosen to reflecta greater morphological similarity to *Dromaeosauripus* than to other didactyl ichnotaxa of inferred dromaeosaurian affinity. However, differences between the two ichnogenera are discussed in the main article text.

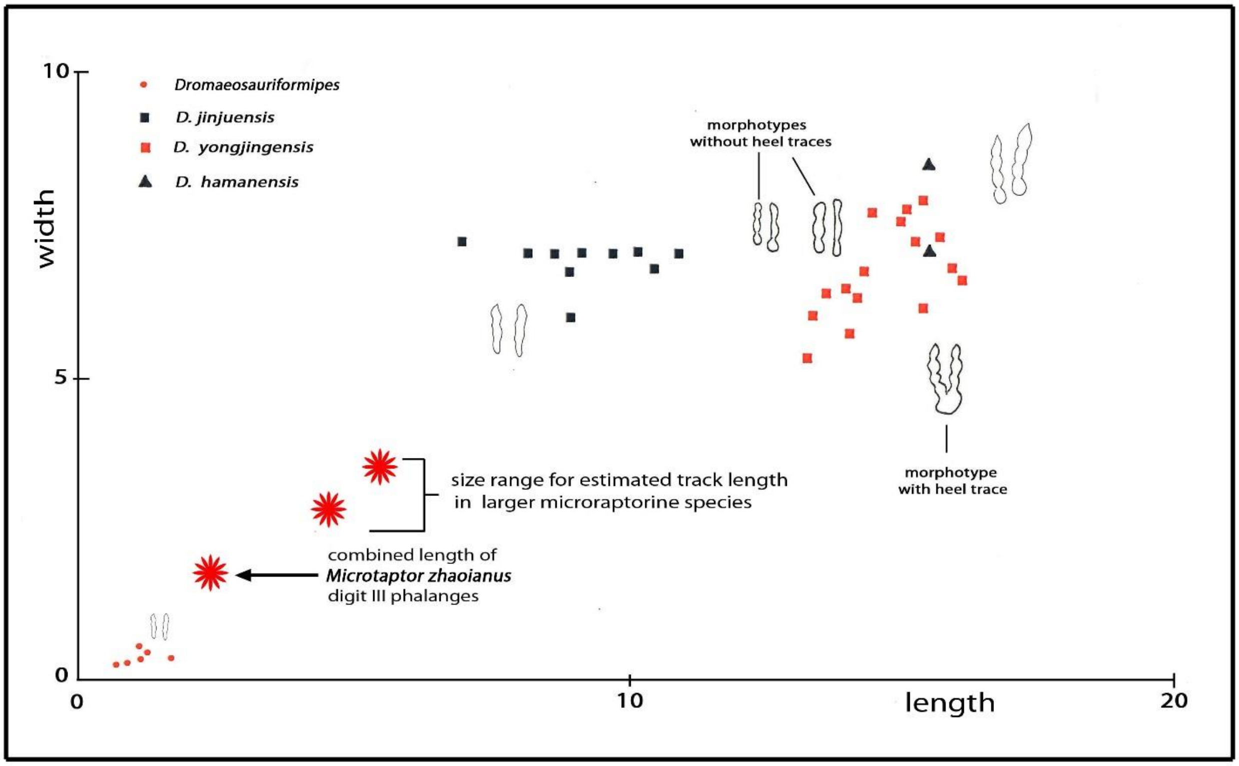

Fig SI 5: Size distribution of large and small dromaeosaur tracks from Cretaceous sites in Korea and China. Note that despite a big size gap between track lengths (~1.0 cm) in the sample of small *Dromaeosauriformipes rarus* ichnogen. et ichnosp. nov. tracks, and the lengths of tracks in the type samples of *D. hamanensis*,[7] *D. jinjuensis* [6] and *D. yongjingensis*, [12] (~10.0 -15.0 cm) the length of digit III in *Microraptor zhaoianus*[31] (red star lower left) would be represented by small tracks about 2.5 cm long. Larger microraptorine species would have lengths in the range of ~ 3.3 - ~ 5.0 cm. Illustrations made by K-S K and M G L in Adobe photoshop (version CS6 www.adobe.com/Photoshop). Compare with Fig. SI 6.

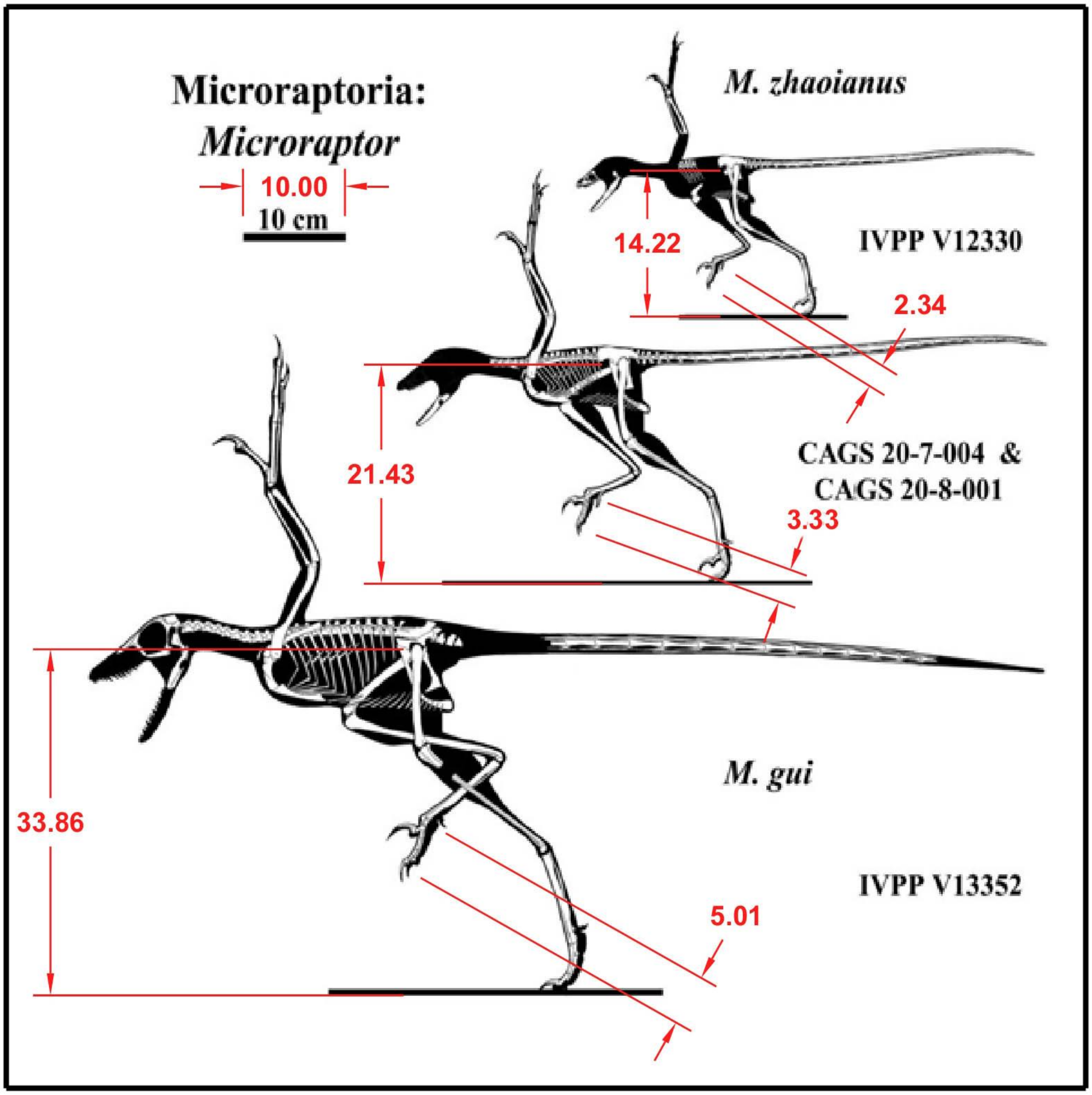

Fig SI 6: Reconstruction of small dromaeosaurid dinosaurs based on well-known microraptorine body fossils to show estimated track lengths derived from foot skeletons. Top, middle and bottom respectively show *M. zhaoianus*,[31] specimen based on Chinese Academy of Geological Sciences (CAGS) specimens (20-7-004 and 20-8-001) and *M. gui*.[32] Compare with Fig. SI 5. Illustrations made by K-S K and M G L in Adobe photoshop (version CS6 www.adobe.com/Photoshop).

4. Ichnotaxonomic considerations

As discussed in the text of the main article, conventions guiding vertebrate ichnotaxonomy emphasize the primacy of morphological difference in the erection of new ichnotaxa[16] and should be independent of inferences about trackmaker identification , geological age or non-morphological criteria. However, morphological differences can be reflection of behavior, which can, in turn, also, be the basis for ichnotaxonomic classification. [SI ref. 3]. A perennial problem arises in considering how much morphological difference exists between species, or traces of different species behavior. In vertebrate ichnology this debate has been ongoing, particularly with regard to theropod tracks. Some authors have proposed that small narrow tracks (ichnogenus *Grallator*) fall on an allometric continuum with larger, wider tracks (ichnogenus *Eubrontes*) and can be "lumped" in the former ichnogenus [SI refs. 4,5]. However, this "lumper" approach has been disputed, due to recognition of size and shape gaps in the perceived continuum,[15] the distribution of small and large morpohotypes in space and time, and the implied lack of trackmaker diversity. Thus, the lumper approach has not been adopted in favor of the "splitter" approach that continues to regard these ichnogenera as distinct and differentiated, [SI ref 6]. even among previous advocates of ichnotaxonomic lumping [SI ref. 7]. The difference between *Dromaeosauriformipes rarus*, described here and previously named *Dromaeosauripus jinjuensis* [6] presents us with a very similar problem, namely that the former ichnospecies is small and narrow and the latter is large and wide. While such differentiated morphologies could theoretically be placed on an allometric continuum, indicating increased breadth with growth, this "lumper" approach would ignore huge size differences, the lack of size and shape continua between the two ichnospecies, and the aforementioned preference for differentiating other well-known ichnospecies with "generally" similar morphologies, but different sizes and distributions in space and time. Thus, on balance, the arguments for ichntaxonomic differentiation of *Dromaeosauriformipes rarus* and *Dromaeosauripus jinjuensis* [6] follow convention and prevail here. These arguments are open to adjustment if and when new ichnological evidence becomes available, especially if it pertains to the size gap and shape differences discussed here.

It should also be noted that the erection of *D. rarus* also follows ichnological conventions and guidelines for acceptable naming of new footprint ichnotaxa. These include multiple common sense criteria, of which the most important are well preserved material, adequate comparison with other similar ichnotaxa, and "a trackway – a series of successive footprints ... [as the] best possible basis for the definition of a footprint ichnospecies." [SI refs 8,9]. Some authors have argued that compound traces made by one individual tracemaker may be named separately, even at the ichnogenus level, under certain

provisions such as “highly significant” behavioral differences [SI ref. 3]: e.g., running v. walking. Thus, potentially trackways T1 and T2 (Fig. SI 3) could be named differently based on estimated trackmaker speed. However, we consider that such differences, are not highly significant in this case, and thus do not warrant such a level of “splitting.”

It is also important to note that quality of preservation is important in the selection of trackways suitable for the definition of new ichnotaxa. In recent years the high abundance and diversity of well-preserved tracks recovered from the Jinju Formation, especially those of small tetrapods [main tex refs[3-5]] indicates that the Jinju Formation represents a good example of a Konservat-Lagerstätten [SI refs. 10, 11], which by definition is a source of material exhibiting superior preservation, as in the case of the *D. rarus* specimens.

5. Speed estimates of small dinosaurian bipeds

There are two well defined trackways (1 and 2) of the presumed small dromaeosaurid, here named *Dromaeosauriformipes rarus*. The holotype trackway 1, (mean FL 10.33 mm), has a mean pace length (PL) of 4.62 cm (PL/FL = 4.47) and an estimated speed of 0.6 m/s (= 2.16 km/hr). By contrast the paratype trackway 2, (mean FL 10.5 mm) has a mean pace length (PL) of 27.8 cm (PL/FL 26.49), giving a speed estimate of 10.5 m/s (= 37.8 km /hr) (main text ref 20). This variation in gait parameters is striking but not unprecedented.

The only trackways made by similar sized diminutive dinosaurs are those named *Minisauripus* (FL size range 11- 60 mm). These tracks are know from trackways with very variable pace lengths (Table SI 1). The smallest track (FL 1.1 cm) has a pace length (PL) of 7.1 cm (PL/FL = 6.45). Some trackways show a lower PL/FL ratio of 3.75. By contrast a similar sized track (FL 1.3 cm) has a pace length of 3.4 cm (PL/FL = 18.0). Thus, the PL/FL range of values from 3.75-18.0 for *Minisauripus* is almost as variable as for *Dromaeosauriformipes rarus* (PL/FL 4.47-26.49). Given that the sample of trackways attributed to very small dinosaurs is relatively small much remains to bel learned about variation in gait and how such gaits compare with those of larger bipedal dinosaurs.

Table SI 1. Foot length (FL) and pace length (PL) parameters for known *Minisauripus* trackways shows considerable variation in PL/FL values. CUE JJ_M01-3 is newly-reported from the Jinju Formkation

**Locality & ref**	**Spec. no**	**FL**	**PL**	**stride**	**PL/FL**	**Speed m/s / km/ hr**	**ref**
Sichuan, China	A 1/2	2.5	16.2	-	6.48	-	8
Sichuan, China	A 4 /12/13	2.7	24.5	-	9.07	-	8
Sichuan, China	A 7/9	2.7	14.0	-	5.18	-	8
Sichuan, China	A 15/16	2.5	18.4	-	7.36	-	8
Shandong, China	A 1/2	5.8	64.5	-	11.12	-	8
Shandong, China	B 1/2	6.3	60.0	-	9.52	-	8
Changseon, Korea	KML 2	3.6	22.5	-	6.25	-	8
Changseon, Korea	CUE 08 (1001)	1.1	7.1	-	6.45	1.18 / 4.25	8
Changseon, Korea	TW1 (1003)	1.4	8.6	18.4	6.14	-	8
Changseon, Korea	TW1 (1002)	1.4	10.8	-	7.71	3.73 /10.36	8
Changseon, Korea	TW2	1.7	21.0	42.1	12.35	-	8
Changseon, Korea	TW3	1.3	15.7	-	12.07	-	8
Changseon, Korea	TW4	1.3	23.4	-	18.0**	-	8
Changseon, Korea	TW5	1.4	18.0	-	12.85	-	8
Changseon, Korea	TW6	1.4	17.8	-	12.71	-	8
Sinsu, Korea	-	1.6	6.0	11.7	3.75*	0.47 / 1.69	8
Yangmozou, China	YMZ-T1	2.6	20.1	40.4	7.73	2.12 /7.73	23
Yangmozou, China	YMZ-T2	2.5	37.5	75.0	15.00	6.24 /22.46	23
Yangmozou, China	YMZ-T3	2.6	27.0	53.8	10.34	3.42 /12.31	23
Jinju Formation Korea	CUE JJ_M01-3.	2.38	20.0	39.6	8.41	2.27/ 8.19	SI ref 11

During the course of the present study the first tracks of ichnogenus *Minisauripus*, well known from the Haman Formation (SI Table 1) were discovered and designated as CUE JJ_M01-3. This specimen, which provides the first evidence of exceptionally well preserved skin impressions (SI ref 11) is here added to the *Minisauripus* database (SI Table 1) as supplemental evidence of small trackmakers and the high quality of track preservation in the Jinju Formation.

SI References

1. Caster, K. A restudy of the tracks of *Paramphibius*. *Journal of Paleontology*, **12**, 3-90 (1938)

2. Hwang, S. H., Norell, M. AS., Qiang, J. and Keqin, G. New specimens of *Microraptor zhaoianus* (theropods: Dromaeosauridae) from Northeastern China. *Noviatates*, 3381, 1-44 (2002)

3. Bertling, M., Braddy, S. J., Bromley, R. G., Demathieu, G. R., Genise, J., Mikula´sˇ, R., Nielsen, J. K., Nielsen, K. S. S., Rindsberg, A. K., Schlirf, M. & Uchman, A. Names for trace fossils: a uniform approach. *Lethaia* , **39**: 265-286. (2006)

4. Olsen P. E. Fossil great lakes of the Newark Supergroup in New Jersey. In: Field Studies of New Jersey Geology and Guide to Field Trips (ed. W. Manspeizer): 352–298. New York State Geol. Assoc., 52nd Ann. Meeting. Rutgers University. (1980)

5. Gierlinski G. New dinosaur ichnotaxa from the Early Jurassic of the Holy Cross Mountains, Poland. Palaeogeography., Palaeoclimatatology Palaeoecology **85**: 137–148. (1991)

6. Lockley, M. G., New perspectives on morphological variation in tridactyl footprints: clues to widespread convergence in developmental dynamics. Geological Quarterly. **53**: 415-432. (2009)

7. Olsen, P. E., Smith, J. B. and Mc Donald, N. G.— Type material of the type species of the classic theropod foot print genera *Eubrontes*, *Anchisauripus* and *Grallator* (Early Jurassic, Hartford and Deerfield basins, Connecticut and Massachusetts, USA). Jornal of Vertebrate Paleontology, 1**8** (3): 586–601. (1998)

8. Sarjeant, W.A.S. "Ten paleoichnological commandment": a standardized procedure for the description of fossil vertebrate footprints. In: *Dinosaur Tracks and Traces* (ed. Gillette, D. D.& Lockley, M. G.) 369-370, Cambridge University Press (1989).

9. Peabody, F. E. Taxonomy and the footprints of tetrapods. *Journal of Paleontology*. 29: 915-918 (1955).

10. Alison, P. Konservat-Lagerstätten: Cause and Classification. *Paleobiology* **14**, 331-344 (1988).

11. Kim, K.S., Lockley, M. G., Lim, J. D., M. G., Xing, L. Exquisitely-preserved, high-definition skin traces in diminutive theropod tracks from the Cretaceous of Korea. *Scientific Reports*. (2018) In review
